# Factors influencing the outcomes of trabeculectomy, conventional canaloplasty, and mitomycin C augmented canaloplasty

**DOI:** 10.1007/s00417-024-06656-x

**Published:** 2024-10-26

**Authors:** Julia Prinz, Matthias Fuest, David Kuerten, Peter Walter, Claus Cursiefen, Verena Prokosch

**Affiliations:** 1 Department of Ophthalmology, Faculty of Medicine and University Hospital of Cologne, 50937 Cologne, Germany; 2 Department of Ophthalmology, RWTH Aachen University, 52074 Aachen, Germany

**Keywords:** Glaucoma, Intraocular pressure, Trabeculectomy, Canaloplasty, Risk factors

## Abstract

**Purpose:**

To compare the efficacy, safety, and factors influencing the outcomes of trabeculectomy (TE), conventional canaloplasty (cCP), and mitomycin C augmented canaloplasty (mCP) in glaucoma patients.

**Methods:**

Intraocular pressure (IOP), the number of IOP-lowering eye drops, and surgery-related complications were evaluated at baseline and through 18 months postoperatively. Correlations between patients’ demographic data, ophthalmic and non-ophthalmic conditions, outcomes and complications were evaluated.

**Results:**

171 patients were included. IOP and IOP-lowering eye drops were significantly (*p* < 0.001) reduced 18 months after TE, cCP, and mCP. At the 18-month follow-up, IOP and IOP-lowering eye drops were significantly lower following TE than cCP (*p* < 0.001, *p* = 0.010, respectively) and mCP (*p* = 0.010, *p* = 0.014). At the 18-month follow-up, complete success rates were significantly higher after TE compared to cCP and mCP for IOP ≤ 21, 18, and 16 mmHg (*p* < 0.001). Qualified success rates for IOP ≤ 16 mmHg were higher following TE than cCP and mCP (*p* = 0.023). In the TE group, clinical hypotony at any postoperative follow-up was positively correlated with previous intravitreal anti-vascular endothelial growth factor (VEGF)-therapy (*p* < 0.001), leukaemia (*p* = 0.002), and a spherical equivalent < -3 dioptres (*p* < 0.001). There were no significant correlations in the cCP and mCP groups.

**Conclusion:**

TE, cCP, and mCP led to a significant reduction in IOP and IOP-lowering eye drops during 18 months of follow-up. At 18 months of follow-up, IOP and IOP-lowering eye drops were significantly lower following TE compared to cCP and mCP. Anti-VEGF-therapy, cystostatic therapy in leukaemia, and a spherical equivalent < -3 dioptres were significantly correlated with postoperative hypotony, macular folds, and choroidal detachment in the TE group.

**Key messages:**

*What is known*

• Trabeculectomy (TE) is considered the gold standard in the surgical management of glaucoma. However, TE involves extensive postoperative management and might be associated with severe surgery-related complications.

*What is new*

• In this study, intraocular pressure (IOP) and IOP-lowering eye drops were significantly lower following TE compared to conventional canaloplasty (cCP) and mitomycin C augmented canaloplasty (mCP) at a follow-up of 18 months.

• In patients undergoing TE, anti-VEGF-therapy, cystostatic therapy in leukaemia, and a spherical equivalent < -3 dioptres were significantly correlated with postoperative hypotony, macular folds, and choroidal detachment.

## Introduction

Glaucoma is a group of chronic neurodegenerative diseases leading to progressive optic nerve damage and irreversible blindness [1, 2]. To date, intraocular pressure (IOP) reduction is the only established treatment option to slow glaucoma progression [3]. When medical or laser treatment fail to control IOP, surgical treatment might be required [4]. Since its introduction by Cairns and Sugar in the 1960s, trabeculectomy (TE) is still considered the gold standard in the surgical management of glaucoma [5–7]. Despite its substantial IOP-lowering effect, TE involves extensive postoperative management and might be associated with severe bleb-related complications, including hypotony, bleb leaks [8], as well as accelerated cataract progression [9], and infection [5, 10].

This has led to the development of less invasive, bleb-independent, and non-penetrating glaucoma surgeries, which efficiently lower IOP while mitigating surgery-related complications [7]. Canaloplasty (CP) is a well-established, non-penetrating, bleb-independent glaucoma surgery [11]. CP combines viscocanalostomy initially described by Stegman et al. in 1995 with a catheterization and placement of an intracanalicular tension suture in Schlemm’s canal over 360 degrees [12]. CP intends to restore the physiological aqueous humour outflow pathway through the trabecular meshwork, Schlemm’s canal, and collector channels to the episcleral veins [13, 14]. Recently, it has been shown that augmenting conventional CP (cCP) by the addition of antimetabolites, such as mitomycin C (MMC; MMC augmented CP, mCP), creates a subconjunctival filtering zone and may provide an additional IOP-lowering effect [15].

Previous studies comparing TE to CP found a higher IOP-lowering effect of TE compared to CP [7]. However, TE was associated with more surgery-related complications compared to CP, mainly including postoperative hypotony [7]. The rate of hypotony varies highly in patients undergoing TE and ranges between 2 and 40% in previous studies [7, 16, 17]. To date, the efficacy, safety, and risk factors for surgical failure of TE compared to cCP and mCP remain to be elucidated.

This study aimed to compare the efficacy and safety of TE, cCP, and mCP with a follow-up of 18 months and to identify potential risk factors for the postoperative outcome and complications.

## Material and methods

### Patient characteristics

In this retrospective study, we included a consecutive series of 88 eyes of 88 patients undergoing TE, 48 eyes of 48 patients undergoing cCP and 37 eyes of 35 patients undergoing mCP.

All patients were treated at the Department of Ophthalmology at the University Hospital of Cologne or Department of Ophthalmology, RWTH Aachen University. The study adhered to the tenets of Helsinki. It was approved by the medical ethics committee of the University of Cologne (21-1286_2) and RWTH Aachen University (EK 015/15, 410/20).

All patients underwent clinical examinations preoperatively (2 weeks to 1 day before surgery) and at 1 month, 3, 6, 12, and 18 months after surgery. The mean deviation of visual field testing (MD), demographic data including age and sex as well as the glaucoma subtype and further health conditions were recorded preoperatively (Table 1). Additionally, all previous eye surgeries were recorded (Table 2). Patients with a prior history of trabeculectomy were excluded from the study. All examinations included measurement of IOP and recording of IOP-lowering eye drops. Additionally, complications were recorded during every follow-up visit.

**Table 1:** Patient characteristics in the trabeculectomy (TE), conventional canaloplasty (cCP), and mitomcycin C augmented canaloplasty (mCP) group. Dpt: Diopter; refractive error calculated as spherical equivalent; POAG: primary open angle glaucoma; PSXG: pseudoexfoliative glaucoma; NTG: normal tension glaucoma; VEGF: vascular endothelial growth factor; *Patients were treated with chlorambucil and vincristine

	TE	cCP	mCP	*p*-value
***n***	88	48	37	
**Age [years]**	68.1 ± 12.0	63.6 ± 12.2	68.1 ± 11.8	0.132
**Patients < 60 years of age**	18 (20.5%)	13 (27.1%)	6 (16.2%)	0.459
**Female**	42 (47.7%)	24 (50.0%)	25 (67.6%)	0.214
**Right eyes**	46 (52.3%)	29 (60.4%)	20 (54.1%)	0.659
**MD [dB]**	‒11.1 ± 9.1	‒10.3 ± 8.9	‒9.9 ± 8.7	0.737
**Spherical equivalent [dpt]**	‒0.8 ± 2.5	‒1.0 ± 2.9	‒1.3 ± 2.1	0.711
**Spherical equivalent < -3 dpt**	12 (13.6%)	9 (18.8%)	4 (10.8%)	0.645
**IOP-lowering eye drops (mean)**	2.5 ± 1.2	2.5 ± 1.0	2.3 ± 1.0	0.667
*-Alpha-Agonists*	*42 (47.7%)*	*28 (58.3%)*	*18 (48.6%)*	*0.430*
*-Beta-Blocker*	*58 (65.9%)*	*31 (64.6%)*	*18 (48.6%)*	*0.143*
*-Carbonic Anhydrase Inhibitors*	*52 (59.1%)*	*31 (64.6%)*	*24 (64.9%)*	*0.743*
*-Prostaglandins*	*63 (71.6%)*	*30 (62.5%)*	*26 (70.3%)*	*0.589*
*-Pilocarpine*	*3 (3.4%)*	*2 (4.2%)*	*1 (2.7%)*	*0.930*
**Glaucoma Subtype**				
POAG	55 (62.5%)	30 (62.5%)	23 (62.2%)	0.999
PSXG	16 (18.2%)	8 (16.7%)	9 (24.3%)	0.724
NTG	5 (5.7%)	2 (4.2%)	1 (2.7%)	0.896
Neovascular Glaucoma	3 (3.4%)	2 (4.2%)	1 (2.7%)	0.553
Angle Closure Glaucoma	2 (2.3%)	1 (2.1%)	0 (0%)	0.381
Pigmentary Glaucoma	3 (3.4%)	1 (2.1%)	2 (5.4%)	0.744
Juvenile Glaucoma	2 (2.3%)	0 (0%)	0 (0%)	0.497
Uveitic Glaucoma	2 (2.3%)	1 (2.1%)	0 (0%)	0.381
Steroid-induced Glaucoma	0 (0%)	3 (6.3%)	1 (2.7%)	0.090
**Further Diagnoses/Conditions**				
Diabetes Mellitus	11 (12.5%)	5 (10.4%)	4 (10.8%)	0.806
Arterial Hypertension	37 (42.0%)	20 (41.7%)	14 (37.8%)	0.989
Peripheral Artery Disease	3 (3.4%)	2 (4.2%)	1 (2.7%)	0.649
Dysthymia	3 (3.4%)	2 (4.2%)	2 (5.4%)	0.924
Thyroid Hypofunction	5 (5.7%)	4 (8.3%)	3 (8.1%)	0.792
Sleep Apnoea	5 (5.7%)	2 (4.2%)	2 (5.4%)	0.483
Leukaemia (Cytostatic Therapy)*	2 (2.3%)	1 (2.1%)	0 (0%)	0.381
Smoker	7 (8.0%)	4 (8.3%)	3 (5.4%)	0.994
Anti-VEGF Therapy	5 (5.7%)	2 (4.2%)	2 (5.4%)	0.930
*-number of intravitreal injections*	12.0 (3–36)	10.5 (9–12)	10.5 (3–18)	‒
*-Retinal Vein Occlusion*	*3 (3.4%)*	*1 (2.1%)*	*1 (2.7%)*	*0.906*
*-Age-related Macular Degeneration*	*2 (2.3%)*	*1 (2.1%)*	*1 (2.7%)*	*0.982*
*-Ranibizumab*	*1 (1.1%)*	*0 (0%)*	*0 (0%)*	*0.620*
*-Bevacizumab*	*3 (3.4%)*	*1 (2.1%)*	*1 (2.7%)*	*0.905*
*-Aflibercept*	*1 (1.1%)*	*1 (2.1%)*	*1 (2.7%)*	*0.810*

**Table 2:** Number and percentages of previous surgeries in the trabeculectomy (TE) and canaloplasty (CP) group. Goniotomy was performed with Kahook® Dual Blade, New World Medical, Rancho Cucamonga, CA, USA, SLT: selective laser trabeculoplasty, CPC: cyclophotocoagulation, CCC: cyclocryocoagulation

Ocular surgery history	TE	cCP	mCP
Phacoemulsification	41 (46.5%)	16 (33.3%)	9 (24.3%)
SLT	12 (13.6%)	5 (10.4%)	8 (21.6%)
Goniotomy	8 (9.1%)	0 (0%)	6 (16.2%)
Canaloplasty	4 (4.5%)	0 (0%)	0 (0%)
Laser Trabeculoplasty	2 (2.3%)	0 (0%)	0 (0%)
Vitrectomy	1 (1.1%)	1 (2.1%)	1 (2.7%)
CPC or CCC	2 (2.3%)	0 (0%)	1 (2.7%)

### Surgical techniques

All surgeries were performed in topical, sub-Tenon, or general anesthesia.

#### TE

A fornix-based conjunctival flap was created at the 12 o’clock position and the conjunctiva and Tenon’s tissue were mobilized. Three non-fragmenting polyvinyl alcohol sponges (ProOphtha Sponges, Lohmann & Rauscher, Neuwied, Germany) soaked with MMC were placed subconjunctivally for 3 min. Then, the MMC application site was washed out with balanced salt solution (BSS, Alcon Pharma GmbH, Freiburg, Germany). A scleral flap of one-third scleral thickness measuring 4 × 4 mm was created. The Trabeculo-Descemet membrane was incised and a surgical sclerostomy of 2 × 2 mm followed by a peripheral iridectomy were performed. The scleral flap was closed with four 10–0 non-absorbable nylon sutures (Ethicon) at its corners and sides. The conjunctiva and Tenon’s tissue were repositioned and closed with Vicryl 10–0 (Ethicon).

#### cCP and mCP

For both cCP and mCP, a conjunctival flap was created at the 12 o’clock limbus position. Unlike in cCP, a sponge soaked with MMC 0.2 mg/mL was inserted subconjunctivally for 3 min in mCP, followed by rinsing with BSS.

In both cCP and mCP, a superficial scleral flap of 4 × 4 mm was then prepared followed by the dissection of a second deep scleral flap measuring 1.5 × 3 mm reaching into the clear cornea and leaving only a few layers over the scleral tissue. The roof of the Schlemm’s canal was detached and the deeper scleral flap was removed. A flexible illuminating microcatheter (iTrackTM250A, iScience Interventional Corporation, Menlo Park, USA) was inserted into the Schlemm’s canal over 360 degrees. Sodium hyaluronate 1.4% (HealonGV, Advanced Medical Optics Inc., Santa Ana, USA) was injected every 2 clock hours into the Schlemm’s canal, guided by the blinking light at the tip of the microcatheter. A 10–0 non-absorbable polypropylene suture (Prolene, Ethicon, Johnson & Johnson Medical Corporation, New Brunswick, USA) was tied to the tip of the microcatheter and threaded through the Schlemm’s canal while the microcatheter was withdrawn from the canal. The polypropylene suture was tightened to apply a moderate tension to the inner wall of Schlemm’s canal. The superficial flap was closed using 10–0 non-absorbable nylon sutures (Ethicon). The conjunctiva was then closed with Vicryl 8–0 (Ethicon).

#### Postoperative management

Postoperatively, patients following TE were treated with prednisolone acetate 1% eye drops (Inflanefran forte, Allergan, Frankfurt am Main, Germany) 8 times daily for 1 week postoperatively, tapering over 6 to 8 weeks. Afterwards, the eye drops were reduced to twice and then once daily for 1 week each. In cases of elevated IOP and flat blebs that inflated with ocular massage, laser suture lysis was performed in the early postoperative period. In patients with signs of scarring of the filtering bleb, including elevated IOP, flattened blebs, or corkscrew vessels, early 5-fluorouracil (5-FU) bleb injections were administered.

Patients following cCP or mCP were treated with prednisolone acetate 1% eye drops 5 times daily for 1 week postoperatively, tapering over 6 to 8 weeks. Afterwards, the eye drops were reduced to twice and then once daily for 1 week each. Pupil dilation was avoided to prevent the formation of anterior synechiae between the iris and the Descemet's window. In patients undergoing mCP, 5-FU bleb injections were considered in patients with elevated IOP values during the early postoperative period.

#### Statistical analysis

The Statistical Package for Social Sciences (IBM SPSS Statistics for Windows, Version 25, Armonk, NY: IBM Corp.) was used for statistical analyses. All values are displayed as mean ± standard deviations (SD). Chi-square tests and Fisher’s exact tests were used for categorical variables. Between-group comparisons for continuous data were performed using one-way repeated measures analysis of variance (ANOVA) with least significant difference (LSD) post-hoc tests. Paired T-tests were used to compare data before and after surgery. Pearson’s correlation test was used for correlation analysis of the following parameters: age, sex, preoperative MD, type of IOP-lowering eye drops preoperatively, glaucoma subtype (primary open-angle glaucoma, pseudoexfoliation glaucoma, normal tension glaucoma, angle closure glaucoma, pigmentary glaucoma, juvenile glaucoma, uveitic glaucoma, steroid-induced glaucoma), anti-vascular endothelial growth factor (VEGF)-therapy, diabetes mellitus, arterial hypertension, peripheral artery disease, dysthymia, thyroid hypofunction, sleep apnoea, leukaemia, smoking status, previous surgery (phacoemulsification, selective laser trabeculeculoplasty, Kahook® goniotomy (Kahook Dual Blade, New World Medical, Rancho Cucamonga, CA, USA), vitrectomy, cyclophotocoagulation, cyclocryocoagulation, 360° trabeculotomy, IOP at 18-month follow-up, number of IOP-lowering eye drops at 18-month follow-up, complete success rate ≤ 21 mmHg, ≤ 18 mmHg, ≤ 16 mmHg, qualified success rate ≤ 21 mmHg, ≤ 18 mmHg, ≤ 16 mmHg. A p-value of < 0.05 was set statistically significant.

## Results

All patients completed the preoperative examination. The 1-, 3-, 6-, 12-, and 18-month follow-up visits were completed by 165 (95.4%), 163 (94.2%), 155 (89.6%), 141 (81.5%), and 135 (78.0%) patients, respectively.

### IOP

**Fig. 1:**
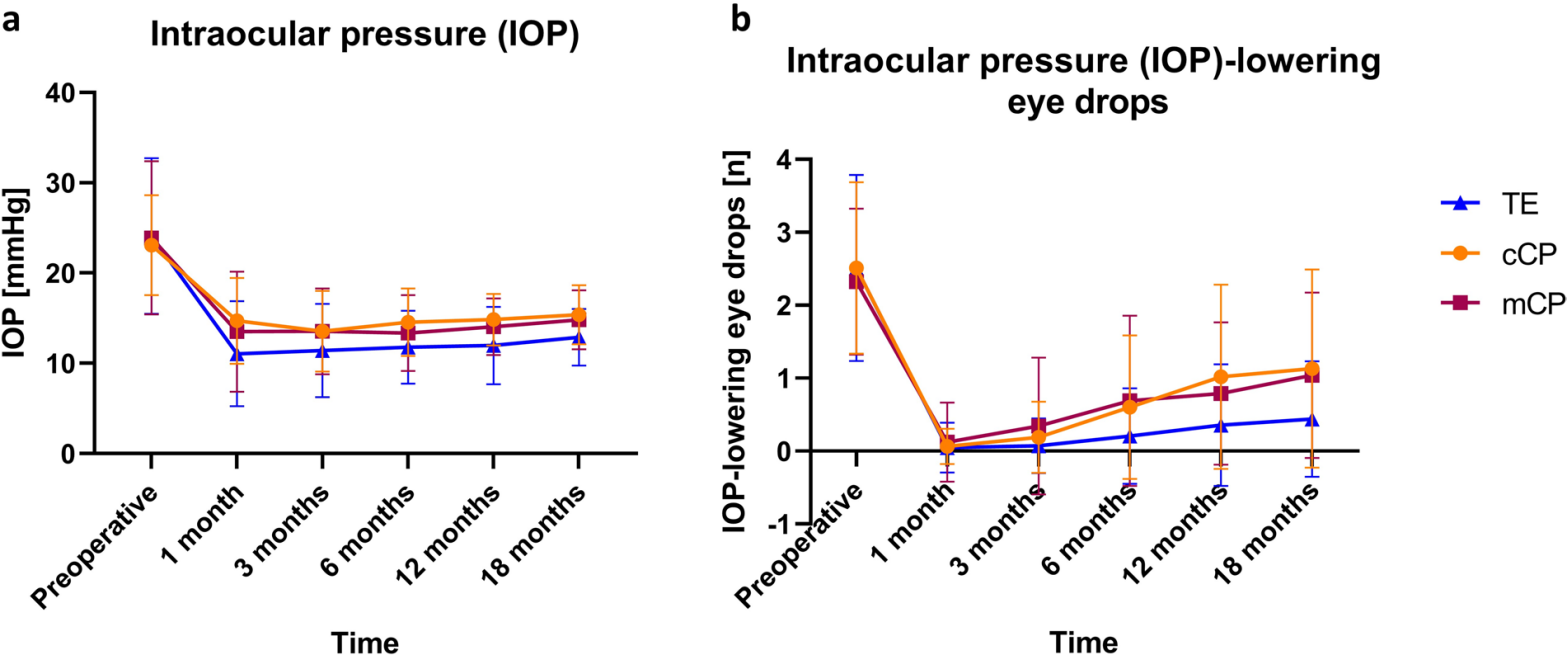
Mean intraocular pressure (IOP, mmHg, **a**) and number of IOP-lowering eye drops (**b**) preoperatively and 1 month, 3, 6, 12, and 18 months following trabeculectomy (TE, blue), conventional canaloplasty (cCP, orange), and mitomycin C augmented canaloplasty (mCP, red)

**Fig. 2:**
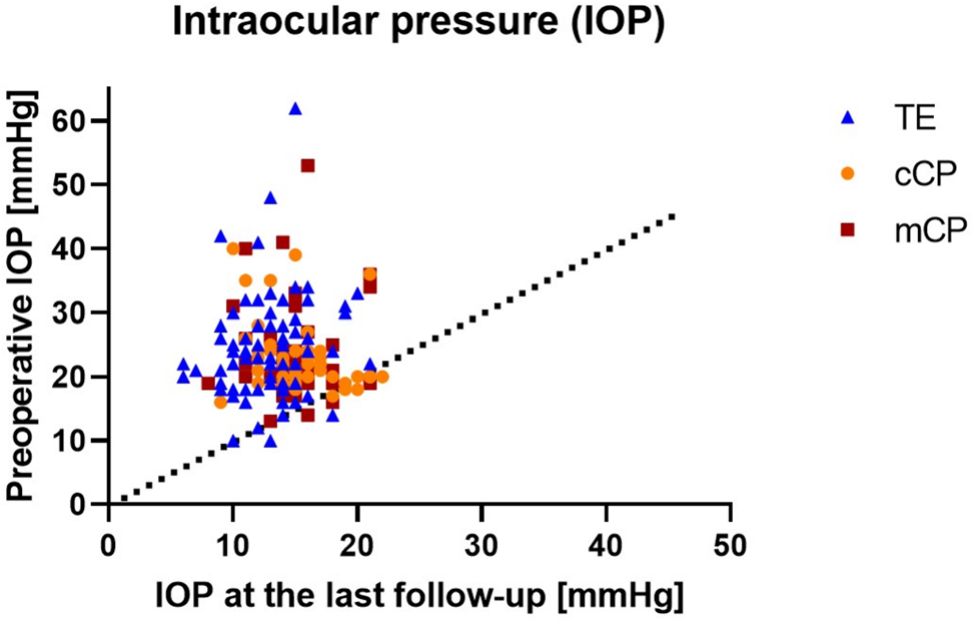
Scatterplot of preoperative versus postoperative intraocular pressure (IOP) values at the last follow-up of each patient. Dots represent eyes that underwent trabeculectomy (TE, blue), conventional canaloplasty (cCP, orange), and mitomycin C augmented canaloplasty (mCP, red)

In patients undergoing TE, IOP was significantly reduced at the 1- (11.1 ± 5.8 mmHg, *p* < 0.001), 3- (11.4 ± 5.2 mmHg, p < 0.001), 6- (11.8 ± 4.0 mmHg, *p* < 0.001), 12- (12.2 ± 4.5 mmHg, *p* < 0.001), and 18-month follow-up (12.9 ± 3.1 mmHg, *p* < 0.001) compared to preoperatively (24.1 ± 8.6 mmHg, Figs. 1a and 2).

Similarly, in the cCP group, IOP was significantly reduced after 1 month (14.7 ± 4.8 mmHg, *p* < 0.001), 3 months (13.6 ± 4.5 mmHg, *p* < 0.001), 6 months (14.6 ± 3.7 mmHg, *p* < 0.001), 12 months (14.8 ± 2.8 mmHg, *p* < 0.001), and 18 months (15.3 ± 3.3 mmHg, *p* < 0.001) compared to preoperatively (23.1 ± 5.5 mmHg).

Compared to pre-mCP (23.9 ± 8.6 mmHg), IOP was significantly reduced at 1 month (13.5 ± 6.7 mmHg, *p* = 0.002), 3 months (13.6 ± 4.8 mmHg, *p* < 0.001), 6 months (13.3 ± 2.8 mmHg, *p* < 0.001), 12 months (14.1 ± 3.1 mmHg, *p* < 0.001), and 18 months (14.9 ± 3.3 mmHg, *p* < 0.001) postoperatively.

At the 1-, 6-, and 12-month follow-up, IOP was significantly lower following TE than cCP (*p* < 0.001, *p* < 0.001, *p* < 0.001, respectively), whereas IOP was similar in the TE and mCP (*p* = 0.055, *p* = 0.088, *p* = 0.051) as well as in the mCP and cCP groups (*p* = 0.379, *p* = 0.259, *p* = 0.439). At the 18-month follow-up, IOP was significantly lower comparing TE to cCP (*p* < 0.001) and comparing TE to mCP (*p* = 0.010; cCP vs. mCP: *p* = 0.652).

### IOP-lowering eye drops

The mean number of IOP-lowering eye drops was significantly reduced 1 month (0.1 ± 0.3, p < 0.001), 3 months (0.1 ± 0.4, p < 0.001), 6 months (0.2 ± 0.7, p < 0.001), 12 months (0.4 ± 0.8, p < 0.001), and 18 months (0.4 ± 0.8, p < 0.001) post-TE compared to preoperatively (2.5 ± 1.3, Fig. 1b).

In patients undergoing cCP, the mean number of IOP-lowering eye drops was significantly reduced 1 month (0 ± 0.2, *p* < 0.001), 3 months (0.2 ± 0.5, *p* < 0.001), 6 months (0.6 ± 1.0, *p* < 0.001), 12 months (1.0 ± 1.3, *p* < 0.001), and 18 months (1.1 ± 1.4, *p* = 0.001) post-cCP compared to preoperatively (2.5 ± 1.0).

Compared to pre-mCP (2.3 ± 1.0), the number of IOP-lowering eye drops was significantly reduced 1 month (0.1 ± 0.5, *p* < 0.001), 3 months (0.3 ± 1.0, *p* < 0.001), 6 months (0.7 ± 1.2, *p* < 0.001), 12 months (0.8 ± 1.0, *p* < 0.001), and 18 months (1.1 ± 1.0, *p* < 0.001) postoperatively).

At the 6-month follow-up, the number of IOP-lowering eye drops was significantly lower following TE than cCP (*p* = 0.013) and comparing TE to mCP (*p* = 0.015; cCP vs. mCP: *p* = 0.679). At 12 months of follow-up, the number of IOP-lowering eye drops was significantly lower in the TE group compared to cCP (*p* < 0.001) and similar after TE vs. mCP (*p* = 0.074) and cCP vs. mCP (*p* = 0.371). 18 months postoperatively, the number of IOP-lowering eye drops was significantly lower following TE than cCP (*p* = 0.001) and comparing TE to mCP (*p* = 0.014; cCP vs. mCP: *p* = 0.853).

### Complete and qualified success

**Fig. 3:**
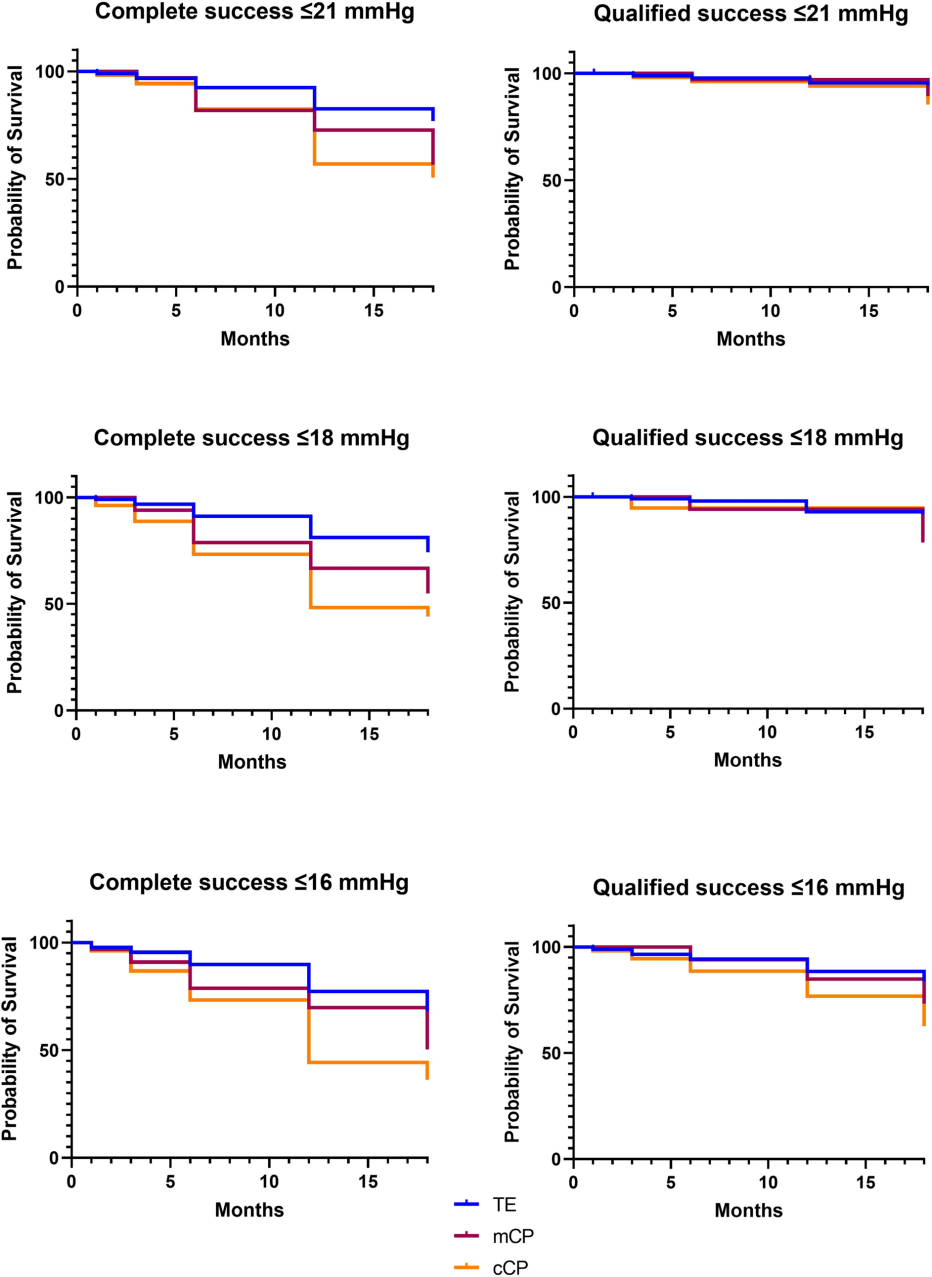
Kaplan Meier curves for complete and qualified success rates (≤ 21 mmHg, ≤ 18 mmHg, ≤ 16 mmHg) during 18 months following trabeculectomy (TE, blue), conventional canaloplasty (cCP, orange), and mitomycin C augmented canaloplasty (mCP, red)

At the 18-month follow-up, complete success rates were 77.0% in the TE, 56.6% in the mCP and 50.6% in the cCP group for IOP ≤ 21 mmHg (*p* < 0.001), 74.2%, 54.9%, and 44.0%, respectively, for IOP ≤ 18 mmHg (*p* < 0.001), and 68.2%, 50.3%, and 36.3% for IOP ≤ 16 mmHg (*p* < 0.001, Fig. 3). Qualified success rates were 94.3% in the TE, 89.3% in the mCP, and 85.4% in the cCP group for IOP ≤ 21 mmHg (*p* = 0.249), 91.8%, 78.4%, and 86.4%, respectively, for IOP ≤ 18 mmHg (*p* = 0.226), and 83.7%, 73.3%, and 62.6% for IOP ≤ 16 mmHg (*p* = 0.023).

### Complications

No relevant intraoperative complications occurred in any group. Intraoperatively, successful 360° catheterization and placement of a suture were achieved in all patients undergoing cCP and mCP.

All postoperative complications and further interventions are summarized in Table 3. Early (within the first 3 postoperative months) numerical hypotony, defined as IOP ≤ 5 mmHg at any follow-up visit, was observed significantly more frequently in patients undergoing TE (20.5%) compared to cCP (8.3%, *p* = 0.010; cCP vs. mCP: *p* = 0.221; TE vs. mCP: *p* = 0.310; Table 3). Clinical hypotony requiring intervention occurred significantly more frequently in patients undergoing TE compared to cCP (*p* = 0.013), whereas there were no significant differences between TE and mCP (*p* = 0.228) or cCP and mCP (*p* = 0.327).

**Table 3:** Postoperative complications and interventions following trabeculectomy (TE), conventional canaloplasty (cCP), and mitomycin C augmented canaloplasty (mCP)

Complications / interventions	TE	cCP	mCP	*p*-value
**Early (≤ 3 months)**				
Numerical Hypotony ≤ 5 mmHg within first 3 months	18 (20.5%)	4 (8.3%)	4 (10.7%)	**0.035**
Clinical Hypotony requiring Intervention	10 (11.4%)	0 (0%)	1 (2.7%)	**0.006**
-Shallow Anterior Chamber	4 (4.5%)	0 (0%)	0 (0%)	0.468
-Macular Folds	5 (5.7%)	0 (0%)	0 (0%)	0.140
-Choroidal Detachment	8 (9.1%)	0 (0%)	0 (0%)	**0.017**
-Descemet Membrane Detachment	0 (0%)	0 (0%)	1 (2.7%)	0.157
Hyphema	2 (2.3%)	16 (33.3%)	14 (37.8%)	** < 0.001**
Corneal Erosion	3 (3.4%)	1 (2.7%)	0 (0%)	0.264
Laser Suture Lysis	18 (20.5%)	0 (0%)	0 (0%)	‒
Blebitis / Endophthalmitis	0 (0%)	0 (0%)	0 (0%)	‒
5-FU Bleb Injection (Patients)	38 (43.2%)	‒	3 (8.1%)	‒
5-FU Bleb Injection (Number)	99	‒	5	‒
**Late (> 3 months)**				
Numerical Hypotony ≤ 5 mmHg > 3 months	3 (3.4%)	0 (0%)	0 (0%)	0.259
Clinical Hypotony requiring Intervention	0 (0%)	0 (0%)	0 (0%)	‒
Scleral Flap Revision	5 (5.7%)	‒	‒	‒
360° Trabeculotomy	‒	5 (10.4%)	2 (5.4%)	‒
Scarring of the Filtering Bleb	14 (15.9%)	‒	0 (0%)	‒
Blebitis / Endophthalmitis	0 (0%)	0 (0%)	0 (0%)	‒
Further Surgical Glaucoma Intervention	3 (3.4%)	4 (8.3%)	3 (8.1%)	0.401

There were no differences in the occurrences of shallow anterior chamber (*p* = 0.468), Descemet membrane detachment (*p* = 0.157), macular folds (*p* = 0.140), or corneal erosion (*p* = 0.264). Hyphema was more frequent after cCP (*n* = 16, 33.3%, *p* = 0.014) and mCP (*n* = 14, 37.8%, *p* = 0.011) than TE.

In the cCP group, 360° trabeculotomy was performed in 5 (10.4%) patients after a mean follow-up of 6.5 ± 0.7 months and in 2 (5.4%) patients at 5 and 9 months following mCP (*p* = 0.411, Table 3).

Laser suture lysis was performed in 18 (20.5%) patients in the TE group. 5-FU bleb injections were performed 99 times in 38 (43.2%) patients after TE and 5 times in 3 patients (8.1%) after mCP. In the TE group, 5 (5.7%) patients required scleral flap revision.

In 2 (2.3%) patients of the TE group, glaucoma drainage implant surgery was performed due to insufficient IOP reduction at 14 and 18 months post-TE. Another patient (1.1%) in the TE group underwent cyclophotocoagulation 15 months after TE. In the cCP group, three patients underwent TE 12 to 18 months post-cCP. Another patient (2.7%) of the cCP group received an ab externo polymer stent (Preserflo® Microshunt, Santen, Osaka, Japan). In the mCP group, 3 patients underwent glaucoma drainage implant surgery 16.0 ± 2.1 months post-mCP.

### Factors influencing postoperative outcomes

All significant correlations between any parameters and outcomes of TE, cCP, and mCP are displayed in Table 4.

**Table 4:** Significant positive correlations of all tested parameters that might influence the outcomes of trabeculectomy (TE), conventional canaloplasty (cCP), and mitomycin C augmented canaloplasty (mCP). VEGF: Vascular Endothelial Growth Factor. Dpt: Diopter

Correlation		Significant *p*-values		
		TE	cCP	mCP
**Numerical Hypotony**	Clincial Hypotony	< 0.001	‒	‒
	Choroidal Detachment	< 0.001	‒	‒
	Macular Folds	0.024	‒	‒
	Age < 60 years	0.004	‒	‒
	Spherical equivalent < -3dpt	< 0.001	‒	‒
	Neovascular Glaucoma	0.004	‒	‒
	Leukaemia	< 0.001	‒	‒
	Anti-VEGF Therapy	< 0.001	‒	‒
**Clinical Hypotony**	Macular Folds	< 0.001	‒	‒
	Numerical hypotony	< 0.001	‒	‒
	Choroidal Detachment	< 0.001	‒	‒
	Anti-VEGF Therapy	< 0.001	‒	‒
	Neovascular Glaucoma	0.026	‒	‒
	Leukaemia	0.002	‒	‒
	Spherical equivalent < -3dpt	0.045	‒	‒
**Macular Folds**	Clinical Hypotony	< 0.001	‒	‒
	Numerical Hypotony	0.024	‒	‒
	Anti-VEGF Therapy	< 0.001	‒	‒
	Leukaemia	0.002	‒	‒
	Spherical equivalent < -3dpt	< 0.001	‒	‒
**Choroidal Detachment**	Clinical Hypotony	< 0.001	‒	‒
	Numerical Hypotony	< 0.001	‒	‒
	Macular folds	< 0.001	‒	‒
	Anti-VEGF Therapy	0.006	‒	‒
	Leukaemia	0.002	‒	‒
	Spherical equivalent < -3dpt	0.045	‒	‒
**Age < 60 years**	Numerical hypotony	0.005	‒	‒
**Scleral Flap Revision**	Leukaemia	0.024	‒	‒
**Anti-VEGF Therapy**	Clinical Hypotony	< 0.001	‒	‒
	Macular Folds	< 0.001	‒	‒
	Choroidal Detachment	0.006	‒	‒
**Leukaemia**	Clinical Hypotony	0.002	‒	‒
	Macular Folds	0.002	‒	‒
	Choroidal Detachment	0.024	‒	‒
	Scleral Flap Revision	0.024	‒	‒
**Spherical equivalent < -3dpt**	Numerical Hypotony	0.005	‒	‒
	Clinical Hypotony	0.045	‒	‒
	Macular folds	< 0.001	‒	‒
	Choroidal Detachment	0.045	‒	‒
**Neovascular Glaucoma**	Clinical Hypotony	0.026	‒	‒
	Numerical Hypotony	0.004	‒	‒
	Anti-VEGF Therapy	< 0.001	‒	‒

## Discussion

According to the main results of the present study, TE, cCP, and mCP are associated with a significant reduction in IOP and IOP-lowering eye drops during 18 months of follow-up. At 18 months of follow-up, IOP and IOP-lowering eye drops were significantly lower following TE compared to cCP and mCP. Intravitreal anti-VEGF-therapy, cytostatic therapy due to leukaemia, and a spherical equivalent < -3 dioptres were positively correlated with postoperative hypotony, macular folds, and choroidal detachment.

At the 18-month follow-up, TE led to lower IOP and a lower number of IOP-lowering eye drops compared to cCP and mCP in this study. Complete success for IOP ≤ 21, 18, and 16 mmHg as well as qualified success rates for ≤ 16 mmHg were higher after TE compared to cCP or mCP. Similarly, previous studies comparing TE to cCP showed higher success rates after TE than cCP [7] and comparing TE to mCP [18]. Likewise, lower IOP values following TE compared to cCP were found in previous comparative studies [19] and meta-analyses [15, 20, 21]. However, some studies reported non-significantly lower IOP levels after TE compared to cCP [7, 22]. Hypothetically, higher preoperative IOP values in the TE groups (24.1 mmHg in our study vs. 28.1 mmHg [22] or 26.3 mmHg [23] in previous studies), proportion of patients receiving postoperative 5-FU bleb injections (43.2% in our study vs. 26.7% in a previous study [22]), and differences in the distribution of glaucoma subtypes might account for differences in IOP outcomes between TE and cCP.

Barnebey investigated the efficacy and safety of mCP and reported an IOP of 13.4 mmHg without the use of IOP-lowering eye drops at the 12-month follow-up. Similarly, in our study, mean IOP was 14.1 mmHg with 0.8 IOP-lowering eye drops 12 months following mCP [24].

To the best of our knowledge, this is the first study comparing TE, cCP, and mCP, and investigating factors that might influence the outcomes of these surgeries.

Previously, myopia, male gender, young age, pseudoexfoliation glaucoma, and the use of antifibrotic agents were described as risk factors for postoperative hypotony and choroidal detachment [25]. Our study confirmed previous findings that myopia was associated with postoperative hypotony. Also, young age < 60 years was correlated with numerical hypotony which is consistent with previous studies [26].

Moreover, our results show that anti-VEGF therapy was significantly correlated with postoperative hypotony, macular folds, and choroidal detachment. In this study, patients receiving anti-VEGF agents were treated with intravitreal injections of ranibizumab, bevacizumab, and aflibercept due to neovascular AMD or retinal vein occlusion. Anti-VEGF agents decrease angiogenesis and delay wound healing [27]. In TE, interruption of the fibroproliferative phase by anti-VEGF agents might lead to less scarring of the Tenon's capsule and the conjunctiva and enhance bleb survival [28]. Previous studies reported on subconjunctival or sub-Tenon application of anti-VEGF agents during TE, however, intravitreal injections of anti-VEGF agents have been shown to be the most effective route of administration for intraocular tissues [29]. Also, repeated intravitreal injections might lead to a cumulative effect [29–31]. An in vivo experimental study showed that subconjunctival injection of bevacizumab after TE resulted in higher and wider blebs and lower IOP levels compared to intravitreal bevacizumab injections or injection of balanced salt solution in New Zealand rabbits [31]. A double-blinded, randomized study found that a single intravitreal bevacizumab dose applied during TE with MMC resulted in a lower IOP during the first postoperative month compared with TE with MMC alone [28]. At the 12-month follow-up, 6% of patients undergoing TE with MMC and single dose adjunctive intravitreal bevacizumab and 17% of patients undergoing TE with MMC alone required IOP-lowering eye drops. Also, patients undergoing TE with MMC and intravitreal bevacizumab postoperatively showed larger volume blebs with less prominent vessel inflammation over the bleb and non-bleb conjunctiva at the first postoperative month [28]. Similar to our results, three cases of postoperative hypotony after glaucoma drainage device surgery [30] or TE in patients treated with anti-VEGF agents due to neovascular AMD [32] were reported recently.

Likewise, cytostatic drug (chlorambucil, vincristine) therapy in patients with leukaemia was significantly correlated with postoperative hypotony, choroidal detachment, and macular folds in patients undergoing TE. It appears plausible that the systemic administration of cytostatic drugs during filtration surgery reaches the ocular tissues and has a similar effect as the commonly used antimetabolites MMC or 5-FU. Therefore, we hypothesise that cytostatic therapy was associated with highly filtering blebs, contributing to postoperative hypotony.

Concluding, we advocate that patients with filtering blebs following TE who require treatment with anti-VEGF agents or cytostatic drugs might benefit from close follow-up investigations. Importantly, we identified no factors influencing the results of cCP or mCP.

This study has some limitations, including its retrospective design. We prospectively collected retrospective data of our patients. However, a certain selection bias might not be excluded. Also, the limited sample sizes and number of patients lost to follow-up possibly restricts conclusions on the outcomes of TE, cCP, and mCP. However, follow-up examinations were often performed by the local ophthalmologists and patients were referred to our clinic in case of re-increase of IOP or the need of re-intervention. Therefore, it may be assumed that most of the patients lost to follow-up experienced an uneventful postoperative course. Another limitation of our study is that no patients treated with cytostatic drugs were included in the mCP group. Thus, we cannot completely rule out that cytostatic drug therapy might affect the outcomes or postoperative hypotony rates of mCP as well.

## Conclusion

TE, cCP, and mCP are associated with a significant reduction in IOP and IOP-lowering eye drops during 18 months of follow-up. At 18 months of follow-up, IOP and IOP-lowering eye drops were significantly lower following TE compared to cCP and mCP. Intravitreal anti-VEGF-therapy and leukaemia were significantly correlated with postoperative hypotony, macular folds, and choroidal detachment. Concluding, patients following TE who require treatment with anti-VEGF agents or cytostatic drugs might benefit from close follow-up investigations.
